# AMPK Activation Reduces Hepatic Lipid Content by Increasing Fat Oxidation In Vivo

**DOI:** 10.3390/ijms19092826

**Published:** 2018-09-19

**Authors:** Marc Foretz, Patrick C. Even, Benoit Viollet

**Affiliations:** 1 INSERM, U1016, Institut Cochin, Département d’Endocrinologie Métabolisme et Diabète, 24, rue du Faubourg Saint Jacques, 75014 Paris, France; 2 CNRS, UMR8104, 75014 Paris, France; 3 Université Paris Descartes, Sorbonne Paris Cité, 75014 Paris, France; 4 UMR PNCA, AgroParisTech, INRA, Université Paris-Saclay, 75005 Paris, France; patrick.even@agroparistech.fr

**Keywords:** AMPK, liver, lipid metabolism, fatty acid oxidation, indirect calorimetry

## Abstract

The energy sensor AMP-activated protein kinase (AMPK) is a key player in the control of energy metabolism. AMPK regulates hepatic lipid metabolism through the phosphorylation of its well-recognized downstream target acetyl CoA carboxylase (ACC). Although AMPK activation is proposed to lower hepatic triglyceride (TG) content via the inhibition of ACC to cause inhibition of de novo lipogenesis and stimulation of fatty acid oxidation (FAO), its contribution to the inhibition of FAO in vivo has been recently questioned. We generated a mouse model of AMPK activation specifically in the liver, achieved by expression of a constitutively active AMPK using adenoviral delivery. Indirect calorimetry studies revealed that liver-specific AMPK activation is sufficient to induce a reduction in the respiratory exchange ratio and an increase in FAO rates in vivo. This led to a more rapid metabolic switch from carbohydrate to lipid oxidation during the transition from fed to fasting. Finally, mice with chronic AMPK activation in the liver display high fat oxidation capacity evidenced by increased [C^14^]-palmitate oxidation and ketone body production leading to reduced hepatic TG content and body adiposity. Our findings suggest a role for hepatic AMPK in the remodeling of lipid metabolism between the liver and adipose tissue.

## 1. Introduction

AMP-activated protein kinase (AMPK) is a phylogenetically conserved serine/threonine protein kinase viewed as a fuel gauge monitoring systemic and cellular energy status which plays a crucial role in protecting cellular function under energy-restricted conditions [1]. AMPK is a heterotrimeric protein consisting of a catalytic α-subunit and two regulatory subunits, β and γ, with each subunit existing as at least two isoforms. AMPK is activated in response to a variety of metabolic stresses that typically change the cellular AMP:ATP ratio caused by increasing ATP consumption or reducing ATP production, as seen following glucose deprivation and inhibition of mitochondrial oxidative phosphorylation as well as exercise and muscle contraction. Activation of AMPK initiates metabolic changes to reprogram metabolism by switching cells from an anabolic to a catabolic state, shutting down the ATP-consuming synthetic pathways and restoring energy balance. This regulation involves AMPK-dependent phosphorylation of key regulators of many important pathways [2,3].

One of the first identified AMPK targets is acetyl CoA carboxylase (ACC), playing a role in the control of fatty acid metabolism via the regulation of malonyl-CoA synthesis [4]. Malonyl-CoA is both a critical precursor of biosynthesis of fatty acids and an inhibitor of fatty acid uptake into mitochondria via the transport system involving carnitine palmitoyl­transferase-1. By inhibiting ACC and lowering the concentration of its reaction product malonyl-CoA, AMPK activation is expected to coordinate the partitioning of fatty acids between oxidative and biosynthetic pathways by increasing fatty acid oxidation (FAO) capacity and inhibiting de novo lipogenesis (DNL), respectively. For these reasons, AMPK has emerged as a promising therapeutic target to treat metabolic disorders that occur in conditions such as nonalcoholic fatty liver disease (NAFLD). There is now literature precedence demonstrating the impact of hepatic AMPK activation in the setting of NAFLD [5]. In addition, recent advances in the development of allosteric and isoform-biased small-molecule AMPK activators have reinforced the potential for the pharmacological activation of AMPK as a treatment modality for hepatic steatosis [6,7,8,9]. Recent evidences showed that regulation of hepatic lipogenesis by AMPK activation mainly resides in the phosphorylation and inactivation of ACC, but not in the control of lipogenic gene expression [7,8,10]. Accordingly, genetic mouse models of hepatic AMPK deficiency and ACC with knock-in phosphorylation mutations confirmed the importance of the activation of AMPK and phosphorylation of ACC for the improvement of fatty liver disease induced by AMPK-activating drugs [7,8,11]. These studies also provided in vitro and in vivo evidence for the contribution of both hepatic FAO and DNL in the reduction of hepatic triglyceride (TG) accumulation mediated through pharmacological AMPK activation. However, one study recently questioned the effect of liver-specific activation of AMPK on FAO rates in vivo [10]. In that study, by using a genetic mouse model expressing in the liver a gain-of-function AMPKγ1 mutant, Woods et al. demonstrated that the effect of hepatic AMPK activation in the protection against hepatic steatosis is largely dependent on the suppression of de novo lipogenesis, but not on the stimulation of hepatic fatty acid oxidation [10]. Therefore, in the present study, we examined the impact of AMPK activation in the liver on hepatic lipid metabolism and determined its effect on FAO rates in vivo, measured by indirect calorimetry.

## 2. Results

**Figure 1:**
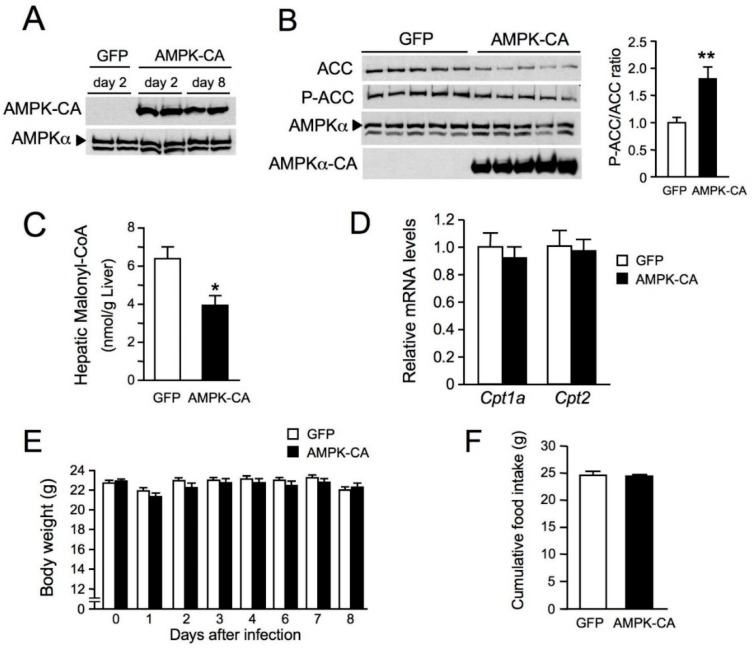
Effects of the expression of an active form of AMP-activated protein kinase (AMPK) in the liver on body weight and food intake. Ten-week-old male C57BL/6J mice received injections of adenovirus (Ad) expressing the green fluorescent protein (GFP) or a constitutively active form of AMPKα2 (AMPK-CA) and were studied for the indicated times after adenovirus injection and in the indicated nutritional state. (**A**) Western blot analysis of liver lysates with antibodies raised against pan-AMPKα and myc-tagged AMPK-CA was performed on days 2 and day 8 after adenovirus administration; (**B**) Western blot analysis of liver lysates from fed mice 48 h after the injection of Ad GFP or Ad AMPK-CA, with the antibodies indicated. Each lane represents a liver sample from an individual mouse. The panel on the right shows Ser79 phosphorylated acetyl CoA carboxylase/ total acetyl CoA carboxylase (P-ACC/ACC) ratios from the quantification of immunoblot images (*n* = 5); (**C**) Hepatic malonyl-CoA levels in 8 h-fasted mice 48 h after the injection of Ad GFP or Ad AMPK-CA (*n* = 5); (**D**) Effect of AMPK activation in the liver on the expression of *Cpt1a* and *Cpt*2 genes. Total RNA was isolated from the liver of 24 h-fasted mice 48 h after the injection of Ad GFP or Ad AMPK-CA (*n* = 6). The expression of *Cpt1a* and *Cpt2* genes was assessed by real-time quantitative RT-PCR. Relative mRNA levels are expressed as fold-activation relative to levels in Ad GFP livers; (**E**) Body weight changes and (**F**) cumulative food intake measured for 8 days after adenovirus administration (*n* = 11–12 per group). Data are means ± standard error of mean (SEM). * *p* < 0.05, ** *p* < 0.01 versus Ad GFP mice by unpaired two-tailed Student’s *t*-test (**B**–**D**,**F**) or by one-way ANOVA with Bonferroni post-hoc test (**E**).

As a first step to elucidating the impact of AMPK activation in the liver on hepatic lipid metabolism in vivo, we generated a mouse model in which AMPK activation specifically in the liver is achieved by expression of a constitutively active AMPK. Mice were injected intravenously with an adenovirus expressing a constitutively active form of AMPKα2 (Ad AMPK-CA) or GFP as a control (Ad GFP). This resulted in AMPK-CA expression restricted to the liver and undetectable in all other tissues (Figure 1A and data not shown). High levels of hepatic AMPK-CA expression were maintained until day 8, with no change in endogenous AMPKα expression (Figure 1A). ACC protein levels were low in Ad AMPK-CA livers, but the phospho-ACC/total ACC ratio was twice that in control livers, demonstrating an increase in ACC phosphorylation and therefore AMPK activation following AMPK-CA expression (Figure 1B). Decreased hepatic malonyl CoA levels in Ad AMPK-CA compared to Ad GFP livers also confirmed inhibition of ACC activity (Figure 1C). The levels of carnitine palmitoyltransferase (CPT)-1a and -2 mRNA expression were similar in the liver of Ad GFP and AMPK-CA mice (Figure 1D). There were no significant changes in body weight and food intake during the week following the injection of AMPK-CA or GFP adenoviruses (Figure 1E,F).

**Figure 2:**
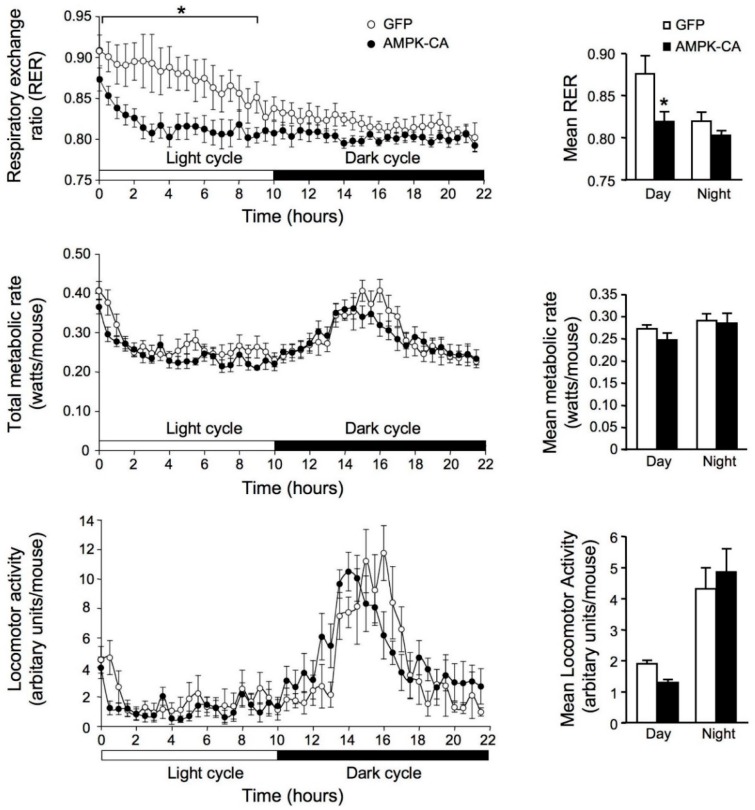
Effects of the expression of an active form of AMPK in the liver on respiratory exchange ratio. Whole-animal indirect calorimetry was used to assess oxygen consumption (VO_2_) and carbon dioxide production (VCO_2_) in mice infected with Ad GFP or Ad AMPK-CA for 48 h. Fed adenovirus-infected mice were placed in a metabolic chamber at 10:00 h. They were kept in the cage for 22 h, with free access to water but no food. Upper panel: The respiratory exchange ratio (RER = VO_2_/VCO_2_) was calculated from VO_2_ and VCO_2_ data and plotted at 15-min intervals. An RER of 1.0 is expected for glucose oxidation and an RER of 0.7 corresponds to lipid oxidation. The right panel shows mean RER results for Ad GFP and Ad AMPK-CA mice (*n* = 6 per group) during light or dark periods. Middle panel: Total metabolic rate. The right panel shows mean metabolic rates for Ad GFP and Ad AMPK-CA mice (*n* = 6 per group) during light and dark periods. Lower panel: Locomotor activity. The right panel shows the mean locomotor activity results for Ad GFP- and Ad AMPK-CA mice (*n* = 6 per group) during light and dark periods. Data are means ± SEM. * *p* < 0.05 versus Ad GFP mice by one-way ANOVA with Bonferroni post-hoc test.

We studied the metabolic consequences of AMPK activation in the liver by monitoring energy expenditure and respiratory exchange ratio (RER) during a 22-h fasting period, determined by indirect calorimetry. The values of RER provide an approximation of carbohydrate and lipid oxidation to generate energy, ranging from 1.0 to approximately 0.7, respectively. In fed mice, the RER associated with the total and resting metabolism rates was lower in Ad AMPK-CA than in Ad GFP mice. During fasting, Ad AMPK-CA mice reached maximal rates of lipid oxidation after only 3 h of fasting, whereas such rates were not achieved until 12 h in Ad GFP mice (Figure 2, upper panel). Thus, AMPK activation in the liver enhances lipid oxidation, leading to a more rapid metabolic switch from carbohydrate to lipid oxidation during the transition from fed to fasting. Thereafter, RER stabilized at the same values in Ad GFP and Ad AMPK-CA mice, suggesting that the rate of lipid oxidation reached the same maximum intensity in both groups. Total and resting metabolic rates and spontaneous activity were similar in Ad GFP and Ad AMPK-CA mice (Figure 2, middle and lower panels). All mice exhibited a period of intense activity during the night period between 00:00 and 06:00 h. According to previous observations of fed mice, this hyperactivity was probably related to the fact that the mice were fasted and seeking for food. Analysis of the changes in total metabolism and RER induced by bursts of spontaneous activity that occurred during the light period (i.e., when RER was lower in Ad AMPK-CA than in control Ad GFP mice) showed that the utilization of glucose and lipids by the working muscles was very similar in both groups (same changes in RER). These observations agree with the conclusion that the rapid mobilization and utilization of lipids in Ad AMPK-CA mice in response to fasting is probably specific to the constitutive activation of AMPK in the liver.

**Figure 3:**
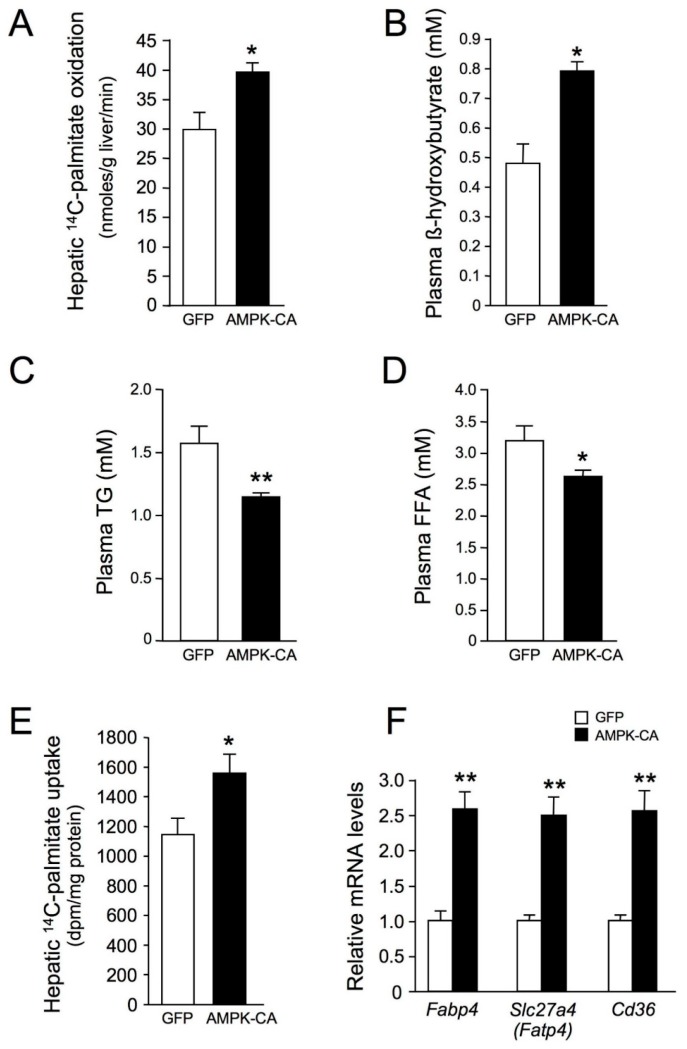
Long-term adenovirus-mediated expression of an active form of AMPK in the liver increases hepatic lipid oxidation and fatty acid uptake. Ten-week-old male C57BL/6J mice received injections of Ad GFP or Ad AMPK CA and were studied at the indicated times after adenovirus injection and in the indicated nutritional state. (**A**) Hepatic [1-^14^C]-palmitate oxidation in fed mice 48 h after the injection of Ad GFP or Ad AMPK-CA (*n* = 4); (**B**) Plasma β-hydroxybutyrate levels in 24 h-fasted mice 48 h after the injection of Ad GFP or Ad AMPK-CA (*n* = 6); (**C**) Plasma triglyceride (TG) and (**D**) plasma free fatty acid (FFA) levels in overnight-fasted mice 8 days after the injection of Ad GFP or Ad AMPK-CA (*n* = 12); (**E**) Hepatic [1-^14^C]-palmitate uptake in 24 h-fasted mice 48 h after the injection of Ad GFP or Ad AMPK-CA (*n* = 5); (**F**) Effect of AMPK activation in the liver on the expression of the fatty acid transporters. Total RNA was isolated from the liver of 24 h-fasted mice 48 h after the injection of Ad GFP or Ad AMPK-CA (*n* = 5). The expression of *Slc27a4* (*Fatp4*), *Cd36*, and *Fabp4* genes was assessed by real-time quantitative RT-PCR. Relative mRNA levels are expressed as fold-activation relative to levels in Ad GFP livers. Data are means ± SEM. * *p* < 0.05, ** *p* < 0.01 versus Ad GFP-infected mice by unpaired two-tailed Student’s *t*-test.

We then investigated whether AMPK activation mediated increased hepatic fatty acid oxidation, by measuring the rate of β-oxidation through assays of [^14^C]-palmitoyl-CoA oxidation in the liver (Figure 3A). Rates of palmitoyl-CoA oxidation were ~25% higher in Ad AMPK-CA mice than in control Ad GFP mice. Indirect support for the increase in FAO is provided by the increase in plasma ketone bodies in Ad AMPK-CA mice (Figure 3B) and a corresponding decrease in plasma triglyceride (TG) and free fatty acid (FFA) concentrations (Figure 3C,D). To determine whether AMPK activation increased fatty acid utilization, [C^14^]-palmitate was injected into Ad AMPK-CA and Ad GFP mice and its incorporation into lipids measured. AMPK activation was associated with an increase in hepatic fatty acid uptake of ~25% (Figure 3E). These findings were correlated with increased expression of the fatty acid transporters *Slc27a4* (fatty acid transport protein 4, *Fatp4*), *Cd36* (fatty acid translocase, *Fat*), and *Fabp4* (fatty acid binding protein 4) (Figure 3F).

**Figure 4:**
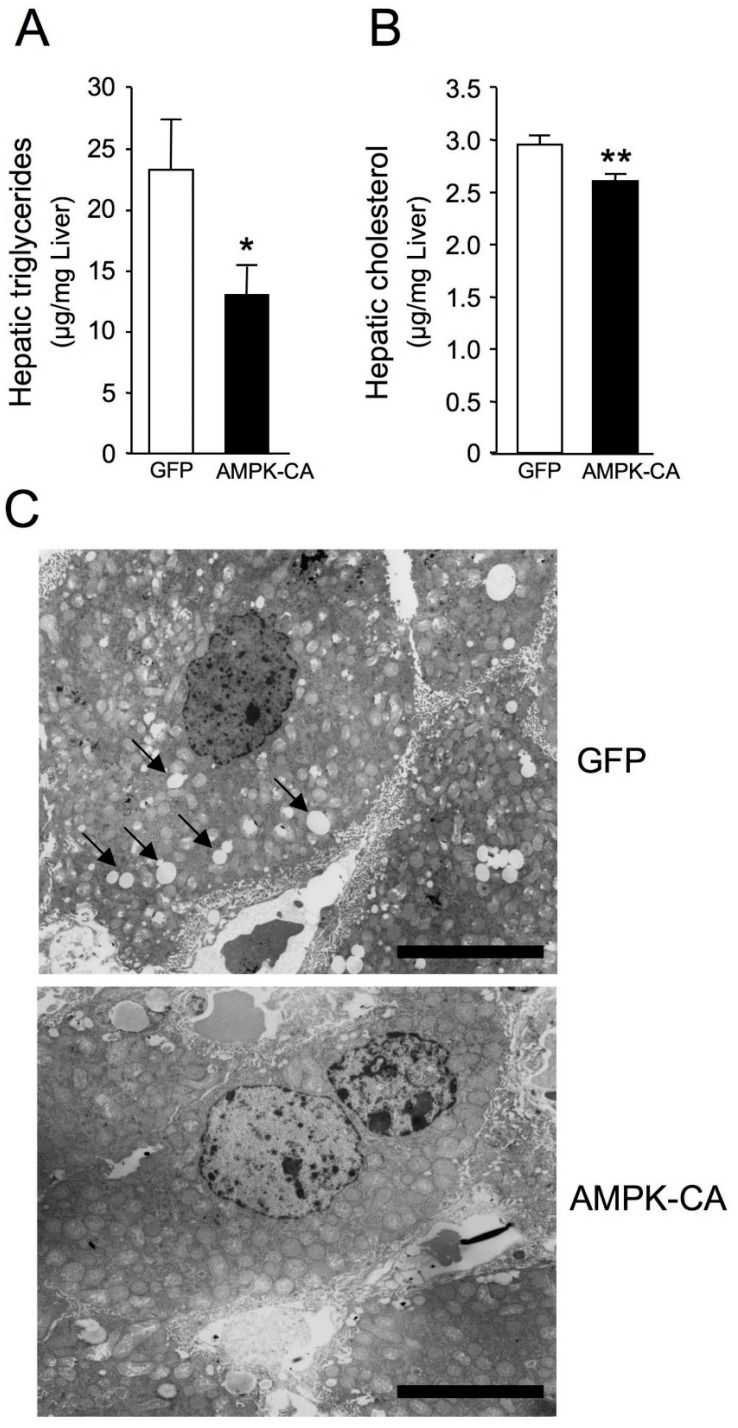
Long-term adenovirus-mediated expression of an active form of AMPK in the liver reduces hepatic lipid accumulation. Ten-week-old male C57BL/6J mice received injections of Ad GFP or Ad AMPK-CA. Fed mice were studied on day 8 after adenovirus administration. (**A**) Liver triglyceride content and (**B**) liver cholesterol content (*n* = 9–10). Data are means ± SEM. * *p* < 0.05, ** *p* < 0.01 versus Ad GFP-infected mice by unpaired two-tailed Student’s *t*-test; (**C**) Representative images of transmission electron microscopy showing the ultrastructure change in Ad GFP and Ad AMPK-CA livers. Scale bar: 10 µm. Black arrowheads in insets depict lipid droplets.

**Figure 5:**
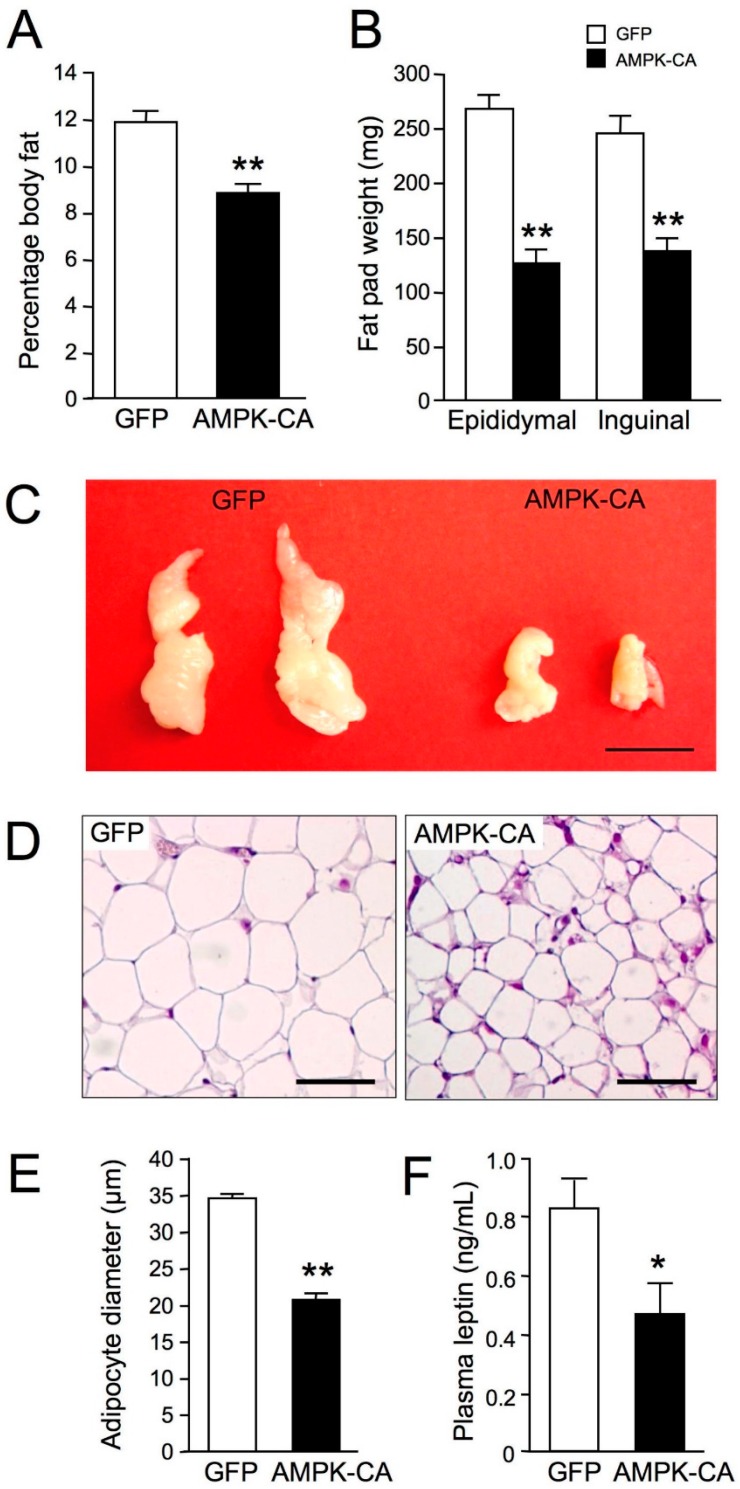
Long-term adenovirus-mediated expression of an active form of AMPK in the liver diminishes peripheral adiposity. Ten-week-old male C57BL/6J mice received injections of Ad GFP or Ad AMPK-CA. Fed mice were studied on day 8 after adenovirus administration. (**A**) Body fat content was measured by dual X-ray absorptiometry (*n* = 10 per group); (**B**) Epididymal and inguinal subcutaneous fat-pad weight (*n* = 10 per group); (**C**) Representative epididymal white fat pads fixed in formalin. Scale bar: 1 cm; (**D**) Representative hematoxylin-and-eosin-stained sections of epididymal adipose tissues. Scale bars: 50 μm; (**E**) Mean adipocyte size in epididymal white adipose tissues. The diameter of at least 200 cells per sample was determined (*n* = 4 mice per group); (**F**) Plasma leptin levels in fed mice (*n* = 10 per group). Data are means ± SEM. * *p* < 0.05, ** *p* < 0.001 versus Ad GFP mice by unpaired two-tailed Student’s *t*-test.

Long-term (8 days) expression of AMPK-CA in liver was sufficient to modify hepatic lipid content and lowered TG levels by ~45% and cholesterol levels by ~10% (Figure 4A,B). This is in line with low abundance of lipid droplets in hepatocytes from Ad AMPK-CA compared to Ad GFP mice revealed by liver ultrastructure changes using transmission electron microscopy (Figure 4C). The increase in hepatic β-oxidation was related to systemic changes in adiposity and resulted in a significant decrease in body fat mass (Figure 5A). This decrease was confirmed by the careful weighing of adipose tissue from epidydimal and inguinal fat pads (Figure 5B). The epidydimal fat pads were much smaller, as was adipocyte diameter (Figure 5C–E). As a result, plasma leptin concentration, a marker of adiposity, was halved in Ad AMPK-CA mice (Figure 5F).

## 3. Discussion

Activation of AMPK has been reported to reduce hepatic lipid content in many preclinical studies, yet the importance of hepatic FAO and DNL for its TG-lowering effect has been unclear [6,7,8,9,10]. Here, we report that AMPK activation in the liver is capable of significant reduction in liver TG through the stimulation of fatty acid utilization, as evidenced by a reduction of RER and increased palmitate oxidation and ketone body production. These results are reminiscent of the acute effect of the direct AMPK activator A-769662, showing a concurrent drop in RER in fed rats and leading to the reduction in liver TGs after chronic treatment of obese mice [9]. Importantly, it has been demonstrated that A-769662 acts in an AMPK-dependent manner to induce fat utilization [12]. We also recently confirmed that A-769662 was capable to restore hepatic fatty acid oxidation after chronic treatment of a fatty liver mouse model [8]. Further support for a significant role of lipid oxidation following hepatic AMPK activation recently came from a study investigating the therapeutic beneficial of the β1-biased activator PF-06409577 in a high-fat-fed mouse model, where the contribution of de novo lipogenesis is essentially negligible for hepatic TG accumulation [7]. Acute or 42 days’ dosing with PF-06409577 resulted in a large increase in circulating β-hydroxybutyrate and lower hepatic TG, an effect that was lost in mice lacking AMPK specifically in the liver [7]. In addition to the impact on FAO, activation of AMPK in the liver has also been largely documented as a source of inhibition of DNL [6,7,8,9,10]. Thus, our results substantially contribute to the current view that following hepatic AMPK activation, lowering of hepatic TGs may arise through the capacity of AMPK to combine between both inhibition of TG synthesis and stimulation of lipid utilization [5,7,8]. Definitive evidence for a dual effect of hepatic AMPK activation on lipid synthesis and utilization is provided by in vitro and in vivo studies with AMPK-deficient mouse models and primary culture of hepatocytes treated with various pharmacological activators of AMPK [7,8]. The balance and contribution between inhibition of DNL and stimulation of FAO may depend on the source of hepatic TGs at the origin of the development of hepatic steatosis. Consistent with this notion, pharmacological AMPK-induced inhibition of DNL has been suggested to play a significant role in the improvement of hepatic steatosis of animal models where DNL mainly contributes to hepatic TG accumulation [7,8]. Similarly, transgenic mice expressing specifically in the liver a naturally occurring gain-of-function AMPKγ1 mutant were completely protected against hepatic steatosis when fed a high-fructose diet, known to increase hepatic lipogenesis [10]. In that study, the effect of AMPK activation was relying exclusively on the inhibition of DNL, because no difference in FAO and RER was detected, despite hepatic AMPK activation. However, it is possible that mice fed a high-sucrose diet preferentially oxidize carbohydrates as their primary source of energy and this obscures the effect of AMPK activation on fat oxidation due to the competition between glucose and fat for substrate oxidation [13]. Intriguingly, in the same study, these mice expressing a gain-of-function AMPKγ1 mutant in the liver failed to stimulate FAO and reduce hepatic lipids when fed a high-fat diet [10]. These results contrast with the effectiveness of the AMPKα2-CA mutant used in the present study and various direct AMPK activators in stimulating hepatic FAO and reducing hepatic TG accumulation in vivo [7,8]. What causes this discrepancy is unclear, but we speculate that basal AMPK activity increased by mutation of the AMPKγ1 subunit is insufficient to fully phosphorylate and inactivate ACC and therefore presumably to stimulate FAO in vivo. Given the observation of the lowering in AMPK activity in the liver of high-fat-fed mice and fatty liver mouse models [8,14,15,16], AMPK activation probably needs to reach a higher threshold before the stimulation of FAO can be effective [8], providing an alternative explanation for the absence of a significant effect on hepatic lipid content in mice expressing the gain-of-function AMPKγ1 mutant on a high-fat diet.

AMPK has been proposed as a potential pharmacological target for the treatment of NAFLD due to its capacity to increase FAO and inhibit DNL in the liver [5]. One mechanism by which AMPK regulates the partitioning of fatty acids between oxidative and biosynthetic pathways is accomplished by the phosphorylation and inactivation of ACC, the rate-controlling enzyme for the synthesis of malonyl-CoA, which is both a critical precursor for biosynthesis of fatty acids and a potent inhibitor of long-chain fatty acyl-CoA transport into mitochondria for β-oxidation. This is supported by the observation of increased fatty acid synthesis and reduced FAO in the liver of mice lacking AMPK phosphorylation sites on ACC1/ACC2 [11]. In addition, these mice are resistant to the inhibition of lipogenesis in vivo induced by the AMPK-activating drugs metformin and A-769662 [11]. The effects of metformin and the direct AMPK activator PF-06409577 on lipid synthesis are abolished in hepatocytes isolated from these mice as well as liver AMPK-deficient mice [7,8,17]. The direct AMPK activator A-769662 also failed to increase fatty acid oxidation in these hepatocytes with mutation at the AMPK phosphorylation sites on ACC isoforms [11]. Thus, the action of AMPK in the improvement of hepatic steatosis is likely mediated through the phosphorylation of ACC to increase FAO and suppress DNL [11]. Recent studies performed in mice and humans treated with pharmacological inhibitors of ACC support the concept that direct inhibition of ACC is a promising therapeutic option for the management of fatty liver disease [18,19].

We have previously shown that short-term (48 h) expression of AMPK-CA in the liver paradoxically induced a concomitant hepatic lipid accumulation and increase in fatty acid oxidation [20]. Interestingly, a similar phenotype is observed during the physiological response to fasting, where hepatic TG contents rise significantly [21]. We hypothesized that in response to short-term AMPK activation, the hepatic lipid oxidation capacity is overloaded by the uptake of mobilized fatty acids from adipose tissue, which are stored temporarily as TGs in the liver until they are oxidized [20]. As anticipated, we report here that long-term (8 days) expression of AMPK-CA finally leads to a decrease in hepatic lipid content, but also in a reduction of body adiposity. However, body weight of Ad AMPK-CA mice was not significantly altered due to the small amount of fat mass loss compared to total body weight. Our data are corroborated with the effect of chronic treatment with the AMPK activators metformin, AICAR, or A-769662 in mice, which are associated with reduced fatty liver and fat pad weight [9,22,23,24], although no change in body composition was reported in diet-induced obese (DIO) mice treated with the AMPK β1-biased activator PF-06409577 [7]. Overall, these observations suggest a role for hepatic AMPK in the remodeling of lipid metabolism through crosstalk between liver and adipose tissue. However, the nature of the hepatic signal triggering the mobilization of fatty acids from adipose tissue to the liver remains to be elucidated. One possibility is the secretion of liver-derived proteins known as hepatokines, which could act on adipose tissue to stimulate lipolysis. FGF21 and Angptl3 are reasonable candidates playing important roles in the regulation of lipid metabolism [25,26]. Interestingly, FGF21 expression is induced by metformin and AICAR in hepatocytes [27].

In conclusion, chronic AMPK activation in the liver increases lipid oxidation, thereby decreasing hepatic lipid content and body adiposity, suggesting a role for hepatic AMPK in the remodeling of lipid metabolism between the liver and adipose tissue. Overall, our data emphasizes the potential therapeutic implications for hepatic AMPK activation in vivo.

## 4. Material and Methods

### 4.1. Reagents and Antibodies

Adenovirus expressing GFP and a myc epitope-tagged constitutively active form of AMPKα2 (AMPK-CA) were generated as previously described [20]. Primary antibodies directed against total AMPKα (#2532), total acetyl-CoA carboxylase (ACC) (#3676), and ACC phosphorylated at Ser79 (#3661) were purchased from Cell Signaling Technology (Danvers, MA, USA) and myc epitope tag (clone 9E10) from Sigma (Saint-Quentin-Fallavier, France). HRP-conjugated secondary antibodies were purchased from Calbiochem (Burlington, MA, USA).

### 4.2. Animals

Animal studies were approved by the Paris Descartes University ethics committees (no. CEEA34.BV.157.12) and performed under a French authorization to experiment on vertebrates (no. 75-886) in accordance with the European guidelines. C57BL/6J mice were obtained from Harlan France (Gannat, France). All mice were maintained in a barrier facility under a 12-h light/12-h dark cycle with free access to water and standard mouse diet (in terms of energy: 65% carbohydrate, 11% fat, 24% protein).

### 4.3. Metabolic Parameters

Blood was collected into heparin-containing tubes, and centrifuged to obtain plasma. Plasma leptin levels were assessed using mouse ELISA kit (Crystal Chem, Elk Grove Village, IL, USA). Plasma triglyceride, free fatty acid, and β-hydroxybutyrate levels were determined enzymatically (Dyasis, Grabels, France).

### 4.4. Liver Triglyceride, Cholesterol, and Malonyl-CoA Contents

For the extraction of total lipids from the liver, a portion of frozen tissue was homogenized in acetone (500 μL/50 mg tissue) and incubated on a rotating wheel overnight at 4 °C. Samples were centrifuged at 4 °C for 10 min at 5000× *g*, and the triglyceride and cholesterol concentrations of the supernatants were determined with enzymatic colorimetric assays (Diasys, Grabels, France). Hepatic malonyl CoA ester content was measured using a modified high-performance liquid chromatography method [28].

### 4.5. Indirect Calorimetry

Mice were placed in a metabolic cage from 10:00 h until 08:00 h the next day (22 h). The metabolic cage was continuously connected to an open-circuit, indirect calorimetry system controlled by a computer running a data acquisition and analysis program, as previously described [29]. Mice were housed with free access to water but no food. Air flow through the chamber was regulated at 0.5 L/min by a mass flow-meter, and temperature was maintained close to thermoneutrality (30 °C ± 1 °C). Oxygen consumption (VO_2_) and carbon dioxide production (VCO_2_) were recorded at one-second intervals. Spontaneous activity was measured by means of 3 piezo-electric force transducers positioned in triangle under the metabolic cage, with sampling of the electrical signal at 100 Hz. Data were averaged every 10 s and stored on a hard disk for further processing. Computer-assisted processing of respiratory exchanges and spontaneous activity signals was performed to extract the respiratory exchanges specifically associated with spontaneous activity (Kalman filtering method) [29]. This separation provided information about total, resting, and activity-related O_2_ consumption and CO_2_ production. The respiratory exchange ratio (RER) was calculated as the ratio of VCO_2_ produced/VO_2_ consumed.

### 4.6. Assessment of Fatty Acid Oxidation in Liver Homogenates

The rate of mitochondrial palmitate oxidation was measured in fresh liver homogenate from fed mice anesthetized with a xylamine/ketamine mixture via intraperitoneal injection according to a modified version of the method described by Yu et al. [30]. The rate of palmitate oxidation was assessed by collecting and counting the radiolabeled acid-soluble metabolites (ASMs) produced from the oxidation of [1-^14^C]-palmitate. Briefly, a portion of liver (200 mg) was homogenized in 19 volumes of ice-cold buffer containing 250 mM sucrose, 1 mM EDTA, and 10 mM Tris–HCl pH 7.4. For the assessment of palmitate oxidation, 75 μL of liver homogenate was incubated with 425 μL of reaction mixture (pH 7.4) in a 25 mL flask. The reaction mixture contained 100 mM sucrose, 10 mM Tris–HCl, 80 mM KCl, 5 mM K_2_HPO_4_, 1 mM MgCl_2_, 0.2 mM EDTA, 1 mM dithiothreitol, 5.5 mM ATP, 1 mM NAD, 0.03 mM cytochrome C, 2 mM L-carnitine, 0.5 mM malate, and 0.1 mM coenzyme A. The reaction was started by adding 120 μM palmitate plus 1.7 μCi [1-^14^C]-palmitate (56 mCi/mmol) complexed with fatty acid-free bovine serum albumin in a 5:1 molar ratio. Each homogenate was incubated in triplicate in the presence or absence of 75 μM antimycin A plus 10 μM rotenone to inhibit mitochondrial β-oxidation. After 30 min of incubation at 37 °C in a shaking water bath, the reaction was stopped by adding 200 μL of ice-cold 3 M perchloric acid. The radiolabeled ASMs produced from the oxidation of [1-^14^C]-palmitate were assayed in the supernatants of the acid precipitate. ASM radioactivity was determined by liquid scintillation counting. Mitochondrial β-oxidation was calculated as the difference between the total β-oxidation rate and the peroxisomal β-oxidation rate, which was determined following incubation of the homogenate with antimycin A and rotenone. Data are expressed in nanomoles of radiolabeled ASM produced per gram of liver per hour.

### 4.7. Palmitate Uptake by the Liver

The in vivo uptake of palmitate by the liver was assessed by injection of 10 μCi [1-^14^C]-palmitate (56 mCi/mmol) complexed with 1% fatty acid-free bovine serum albumin in a final volume of 200 μL PBS via the inferior vena cava in anesthetized 24 h-fasted mice by the intraperitoneal injection of a xylamine/ketamine mixture. Four minutes after injection, the superior vena cava was clamped and the hepatic portal vein was sectioned. A needle was inserted into the inferior vena cava toward the liver, and 10 mL of ice-cold PBS was injected under pressure with a syringe. At the end of this procedure, the liver was pale and the fluid emerging from the portal vein was clear. The liver was removed and used for lipid extraction and for the measurement of radioactivity by scintillation counting. Rates of palmitate uptake are expressed as disintegrations per minute (dpm) per gram of protein per hour.

### 4.8. Fat Mass and Histomorphometry

The total body fat content of mice was determined by dual energy X-ray absorptiometry (Lunar PIXImus2 mouse densitometer; GE Healthcare, Chicago, IL, USA), in accordance with the manufacturer’s instructions. Body weight was determined and the left and right epididymal and inguinal white fat pads were harvested and weighed. Epididymal fat pads were then fixed in 10% neutral buffered formalin and embedded in paraffin. Tissues were cut into 4-μm sections and stained with hematoxylin and eosin. For the determination of adipocyte size, photomicrographs of the stained sections were obtained at ×100 magnification. Mean adipocyte diameter was calculated from measurements of at least 200 cells per sample.

### 4.9. Injection of Recombinant Adenovirus

Male C57BL/6J mice were anesthetized with isoflurane before the injection (between 9:00 and 10:00 h) into the penis vein of 1 × 10^9^ pfu of either Ad GFP or Ad AMPK-CA in a final volume of 200 μL of sterile 0.9% NaCl. Mice were sacrificed 48 h or 8 days after adenovirus injection, as indicated in the figure legends. For the eight-day studies, mouse weight and food intake were measured daily.

### 4.10. Isolation of Total mRNA and Quantitative RT-PCR Analysis

Total RNA from mouse liver tissue was extracted using Trizol (Invitrogen, Carlsbad, CA, USA), and single-strand cDNA was synthesized from 5 µg of total RNA with random hexamer primers (Applied Biosystems, Foster City, CA, USA) and Superscript II (Life Technologies, Carlsbad, CA, USA). Real-time RT-PCR reactions were carried out in a final volume of 20 µL containing 125 ng of reverse-transcribed total RNA, 500 nM of primers, and 10 µL of 2× PCR mix containing Sybr Green (Roche, Meylan, France). The reactions were performed in 96-well plates in a LightCycler 480 instrument (Roche) with 40 cycles. The relative amounts of the mRNAs studied were determined by means of the second-derivative maximum method, with LightCycler 480 analysis software and 18S RNA as the invariant control for all studies. The sense and antisense PCR primers used, respectively, were as follows: for *Cpt1a*, 5′-AGATCAATCGGACCCTAGACAC-3′, 5′-CAGCGAGTAGCGCATAGTCA-3′; for *Cpt2*, 5′-CAGCACAGCATCGTACCCA-3′, 5′-TCCCAATGCCGTTCTCAAAAT-3′; for *Cd36*, 5′-TGGCTAAATGAGACTGGGACC-3′, 5′-ACATCACCACTCCAATCCCAAG-3′; for *Slc27a4* (*Fatp4*), 5′-GCACACTCAGCCGCCTGCTTCA-3′, 5′-TCACAGCTTCTCCTCGCCTGCCTG-3′; for *Fabp4*, 5′-GTGATGCCTTTGTGGGAACCT-3′, 5′-ACTCTTGTGGAAGTCGCCT-3′; for 18S, 5′-GTAACCCGTTGAACCCCATT-3′, 5′-CCATCCAATCGGTAGTAGCG-3′.

### 4.11. Western Blot Analysis

After the indicated incubation time in the figure legends, cultured hepatocytes were lysed in ice-cold lysis buffer containing 50 mM Tris, pH 7.4, 1% Triton X-100, 150 mM NaCl, 1 mM EDTA, 1 mM EGTA, 10% glycerol, 50 mM NaF, 5 mM sodium pyrophosphate, 1 mM Na_3_VO_4_, 25 mM sodium-β-glycerophosphate, 1 mM DTT, 0.5 mM PMSF and protease inhibitors (Complete Protease Inhibitor Cocktail; Roche). Lysates were sonicated on ice for 15 s to shear DNA and reduce viscosity. The tissues were homogenized in ice-cold lysis buffer using a ball-bearing homogenizer (Retsch, Eragny, France). The homogenate was centrifuged for 10 min at 10,000× *g* at 4 °C, and the supernatants were removed for determination of total protein content with a BCA protein assay kit (Thermo Fisher Scientific, Waltham, MA, USA). Fifty micrograms of protein from the supernatant were separated on 7.5% or 10% SDS-PAGE gels and transferred to nitrocellulose membranes. The membranes were blocked for 30 min at 37 °C with Tris-buffered saline supplemented with 0.05% NP40 and 5% nonfat dry milk. Immunoblotting was performed following standard procedures, and the signals were detected by chemiluminescence reagents (Thermo). X-ray films were scanned, and band intensities were quantified by Image J (NIH) densitometry analysis.

### 4.12. Transmission Electron Microscopy

Livers were fixed in 3% glutaraldehyde, 0.1 M sodium phosphate buffer (pH 7.4) for 24 h at 4 °C, postfixed with 1% osmium tetroxide, dehydrated with 100% ethanol, and embedded in epoxy resin. For ultrastructure analysis, ultrathin slices (70–100 nm thick) were cut from the resin blocks with a Reichert Ultracut S ultramicrotome (Reichert Technologies, Depew, NY, USA), stained with lead citrate and uranyl acetate, and examined in a transmission electron microscope (model 1011; JEOL, Tokyo, Japan) at the Cochin Institute electron microscopy facility.

### 4.13. Statistical Analysis

Results are expressed as means ± standard error of mean (SEM). Comparisons between groups were made by unpaired two-tailed Student’s *t*-test or ANOVA for multiple comparisons where appropriate. Differences between groups were considered statistically significant when *p* < 0.05.
